# Height and body mass index in molecularly confirmed Silver–Russell syndrome and the long‐term effects of growth hormone treatment

**DOI:** 10.1111/cen.14715

**Published:** 2022-03-21

**Authors:** Oluwakemi Lokulo‐Sodipe, Eloïse Giabicani, Ana P. M. Canton, Nawfel Ferrand, Jenny Child, Emma L. Wakeling, Gerhard Binder, Irène Netchine, Deborah J. G. Mackay, Hazel M. Inskip, Christopher D. Byrne, I. Karen Temple, Justin H. Davies

**Affiliations:** ^1^ Human Development and Health Faculty of Medicine University of Southampton Southampton UK; ^2^ Department of Paediatric Endocrinology University Hospital Southampton NHS Foundation Trust Southampton UK; ^3^ INSERM, UMR_S 938—Centre de Recherche Saint Antoine, APHP, Hôpital Armand Trousseau, Explorations Fonctionnelles Endocriniennes Sorbonne Université Paris France; ^4^ Division of Endocrinology & Metabolism, Development Endocrinology Unit, Laboratory of Hormones and Molecular Genetics/LIM42, Clinical Hospital, Sao Paulo Medical School University of Sao Paulo Sao Paulo Brazil; ^5^ Pediatric Endocrinology University Children's Hospital Tübingen Germany; ^6^ Child Growth Foundation Sutton Coldfield UK; ^7^ Great Ormond Street Hospital for Children NHS Foundation Trust London UK; ^8^ Wessex Clinical Genetics Service University Hospital Southampton NHS Foundation Trust Southampton UK; ^9^ Wessex Regional Genetics Laboratory Salisbury Hospital NHS Foundation Trust Salisbury UK; ^10^ MRC Epidemiology Unit Faculty of Medicine University of Southampton Southampton UK; ^11^ Cancer Sciences Faculty of Medicine University of Southampton Southampton UK; ^12^ NIHR Southampton Biomedical Research Centre University Hospital Southampton NHS Foundation Trust Southampton UK; ^13^ Present address: Oluwakemi Lokulo‐Sodipe, Oluwakemi Lokulo‐Sodipe, Department of Paediatric Endocrinology, Oxford University Hospitals NHS Foundation Trust John Radcliffe Hospital, Headley Way Oxford UK

**Keywords:** body mass index, growth hormone, height, Silver–Russell syndrome, weight

## Abstract

**Objective:**

Silver–Russell syndrome (SRS) causes short stature. Growth hormone (GH) treatment aims to increase adult height. However, data are limited on the long‐term outcomes of GH in patients with molecularly confirmed SRS. This study evaluated height, body mass index (BMI) and GH treatment in molecularly confirmed SRS.

**Design:**

An observational study with retrospective data collection.

**Patients:**

Individuals with molecularly confirmed SRS aged ≥13 years.

**Measurements:**

Data were collected on height, height gain (change in height standard deviation score [SDS] from childhood to final or near‐final height), BMI and gain in BMI (from childhood to adulthood) and previous GH treatment.

**Results:**

Seventy‐one individuals (40 female) were included. The median age was 22.0 years (range 13.2–69.7). The molecular diagnoses: H19/IGF2:IG‐DMR LOM in 80.3% (57/71); upd(7)mat in 16.9% (12/71) and IGF2 mutation in 2.8% (2/71). GH treatment occurred in 77.5% (55/71).

Total height gain was greater in GH‐treated individuals (median 1.53 SDS vs. 0.53 SDS, *p* = .007), who were shorter at treatment initiation (−3.46 SDS vs. −2.91 SDS, *p* = .04) but reached comparable heights to GH‐untreated individuals (−2.22 SDS vs. −2.74 SDS, *p* = .7). In GH‐treated individuals, BMI SDS was lower at the most recent assessment (median −1.10 vs. 1.66, *p* = .002) with lower BMI gain (2.01 vs. 3.58, *p* = .006) despite similar early BMI SDS to GH‐untreated individuals (median −2.65 vs. −2.78, *p* = .3).

**Conclusions:**

These results support the use of GH in SRS for increasing height SDS. GH treatment was associated with lower adult BMI which may reflect improved metabolic health even following discontinuation of therapy.

## 1 INTRODUCTION

Silver–Russell syndrome (SRS) is a condition characterized by pre‐and postnatal growth failure resulting in small‐for‐gestational‐age (SGA) at birth, short stature, body asymmetry, relative macrocephaly at birth, a protruding or prominent forehead and feeding difficulties during childhood. The diagnosis of SRS can be made using the recently proposed Netchine–Harbison clinical scoring system (NHCSS).^1^, ^2^

In 50–60% of cases, loss of methylation at the intergenic *H19/IGF2* (*H19/IGF2* LOM) differentially methylated region (DMR) at 11p15.5 has been identified.^3^, ^4^ In 5%–10% of cases maternal uniparental disomy of chromosome 7 (upd(7)mat) has been detected.^4^, ^5^ Mutations in *CDKN1C*, *IGF2* and the *PLAG1/HMGA2* pathway are sporadic causes of SRS.^2^ Where there is a clinical diagnosis (i.e., at least the 4/6 of items of the NHCSS) without molecular confirmation and a differential diagnosis has been excluded, the term ‘clinical SRS' is used.^2^ There is considerable overlap with other imprinting disorders, such as Temple syndrome^1^ and maternal uniparental disomy of chromosome 20.^1^ Molecular genetic testing can therefore be useful to confirm a clinical diagnosis.

Children with SRS who are born SGA and remain short can be treated with growth hormone (GH) to increase adult height but there is variation in its use, nationally and internationally. Recent international consensus advocated early GH treatment in SRS for diminishing the risk of hypoglycemia, improving height and optimizing body composition.^2^

GH treatment of SRS increases height velocity^6^ and height standard deviation score (SDS).^7^, ^8^, ^9^ Height gain was inversely related to height^8^, ^10^ and age^7^, ^10^ at treatment initiation. Height at the onset of puberty^8^ and duration of treatment^6^, ^9^, ^10^ were also positively related to height gain. A greater target height SDS positively affects the height gained.^9^ GH treatment is associated with increased final height SDS, which also positively correlated with height at the start of treatment.^9^, ^10^, ^11^ Two studies found that in GH‐treated individuals, males reached a greater final height SDS than females.^8^, ^10^

However, all of these studies included individuals with both molecularly confirmed SRS and clinical SRS and many did not include details of treatment for bone age advancement during puberty, which untreated may compromise final height in SRS.

In association with feeding difficulties in SRS, an extremely lean appearance has been reported in clinical and molecularly confirmed SRS cases.^12^ Evaluation of body mass index (BMI) has been reported in three studies of children with SRS: one study of molecularly and clinically diagnosed SRS reported a mean BMI SDS of −2.2 (SD 1.2).^4^ Another study of molecularly confirmed SRS, which included GH treatment in 69%, reported an overall mean baseline BMI SDS of −2.4 (SD 0.8).^13^ In the third study, examining the effect of appetite stimulation in molecularly and clinically diagnosed SRS, 8.8% had received GH and the median baseline BMI SDS was −2.8.^14^ Some individuals were included in more than one study cohort, they reported BMI at a single time point or before and after a short‐term intervention, and the effect of GH on BMI was not evaluated, but they demonstrate that BMIs in SRS are generally low in childhood. There is less information in adults. In a recent case series of seven adults with molecularly confirmed SRS, the BMI SDS ranged from −2.8 to 2.5 (corresponding to BMI of 16.3–32.3 kg/m^2^), providing some evidence that BMI increases considerably in adulthood in some individuals. Two of the seven (28.6%) had been treated with GH but there was no comparison of BMI between GH treatment groups.^15^ A study of 29 molecularly confirmed and clinical SRS cases treated with GH found mean weight‐for‐height SDS of −2.76 (SD 1.1) at the start of GH and −0.30 (1.1) at the end of treatment (mean age at treatment end 15.7 (SD 1.5) years). Fat mass percentage SDS was −0.51 (SE 0.3) at the start of GH treatment and increased both during treatment and 6 months after treatment discontinuation but subsequently stabilized. BMI SDS was not reported.^16^

There is increasing interest in the long‐term outcome of individuals with SRS in relation to height,^16^ metabolic health,^9^, ^15^ ‘lived experience'^17^ and recently the adult phenotype has been described.^18^ We report the first study comparing long‐term outcomes of GH treatment versus no treatment on height and BMI in a cohort of exclusively molecularly confirmed SRS cases.

## 2 MATERIALS AND METHODS

### 2.1 Study design

The inclusion criteria for the study were: molecularly confirmed SRS (*H19/IGF2* LOM, upd(7)mat and *IGF2* mutations) and age ≥13 years. Cohorts of individuals with molecularly confirmed SRS from the United Kingdom, France and Germany were identified. Data on the German cohort^10^, ^19^ and the UK cohort have previously been reported.^18^ For UK participants, Research and Development approval was granted at University Hospital Southampton (study sponsor) and the NIHR UK Rare Genetic Disease Research Consortium Agreement (‘Musketeers' memorandum') at other genetics centers in the United Kingdom. Ethics approval was granted by the NHS Research Ethics Committee South Central—Hampshire B (REC reference^13^/SC/0630). Ethics approval in France was granted by written informed consent for participation received either from the patients themselves or their parents, in accordance with French national ethics rules (Assistance Publique—Hôpitaux de Paris authorization no. 681). Ethics approval in Germany was granted by the Ethical Committee of the Medical Faculty of the University of Tübingen.

### 2.2 Growth assessment

Height and weight measurements were documented at a single study visit (UK participants) or from case note review of the most recent follow‐up appointment (participants from France and Germany). Height was measured using a stadiometer (free‐standing in the United Kingdom and France; wall‐mounted in Germany). Measurements (including age, height and height SDS) and intervention for growth including GH, gonadotrophin‐releasing hormone analogs (GnRHa) and aromatase inhibitors were obtained from medical records. Final height was defined as: (1) height velocity <0.5 cm/year; (2) height at age ≥18 years or (3) as reported in the medical records. Near final height was defined as chronological age and bone age >16 years (as determined by local standard practice). BMI was calculated as weight (kg) divided by height (m) squared. Target height was calculated as: (maternal height [cm] + paternal height[cm])/2 with 6.5 cm subtracted for female participants and 6.5 cm added for male participants.^20^ SDS were calculated for all heights and weights using the age‐ and country‐specific reference data. The growth reference data for the UK participants were the UK 1990 standard^21^; for the French cohort, Usher and McLean^22^ were used for the birth data and Sempé et al.^23^ and Rolland‐Cachera et al.^24^ for BMI; and, for the German cohort, Prader et al.^25^ and Niklasson et al.^26^ were used. Where the age of the individual was greater than the upper age limit, the data for the maximum age available was used.

Early height and weight data were defined as before the start of GH treatment in the GH‐treated group and as close to age 2–5 years as available in the GH‐untreated group. These data were used to calculate early height SDS and early BMI SDS. Total height gain was calculated as the difference between current height SDS and early height SDS. Change in BMI SDS was calculated similarly as the difference between BMI SDS at the most recent evaluation and early BMI SDS.

### 2.3 Molecular testing

Molecular genetic testing was performed on genomic DNA extracted from peripheral blood leukocytes. Methylation‐specific polymerase chain reaction (MS‐PCR) and methylation‐specific multiplex ligation‐dependent probe amplification (MS‐MLPA) were performed as previously reported.^27^, ^28^ Molecular testing of the French patients was performed after sodium bisulfite treatment of DNA, by TaqMan Allele‐Specific Methylated Multiplex Real‐Time Quantitative PCR (ASMM RTQ‐PCR) as previously described.^29^ Molecular testing of the German cohort was performed with MS‐MLPA after bisulfite treatment of DNA and disomies were confirmed with single‐nucleotide polymorphism microarray as previously described.^30^

### 2.4 Statistical analyses

The overall cohort was categorized on the basis of any prior GH treatment. Various characteristics of GH‐untreated and GH‐treated cases were compared, including early height SDS. Mann–Whitney *U* tests were used to compare continuous variables between two groups (i.e., GH‐treated vs. GH‐untreated or GH pre‐ vs. post‐age 4 years) and the Kruskal–Wallis test for comparison between three groups (i.e., United Kingdom, French and German cohorts or GH‐/GnRHa‐untreated, GH‐treated GnRHa‐untreated and GH‐/GnRHa‐treated). Associations between categorical variables were tested using Fisher's exact test. Statistical significance was initially set as *p* < .05. However, in line with recent discussion, *p* values were not considered purely dichotomously (i.e., significant vs. not significant).^31^ Univariate analysis of variance/multiple linear regression was performed with height SDS as the dependent variable, GH treatment as a fixed factor and covariates of sex, epigenotype and target height SDS. Height gain was assessed by adjustment for early height SDS. Regressing change in SDS on early height would not be appropriate as it is a biased method leading to regression to the mean.^32^ Similar models were used to examine BMI at the latest assessment and BMI change. The country of origin of the cohort, age of puberty onset and GnRHa treatment did not contribute to the final models. Data analysis was performed using SPSS Statistics version 24 (International Business Machines Corporation).

## 3 RESULTS

### 3.1 Participants

A total of 71 individuals (40 females) were included (33 from the United Kingdom; 17 from France and 21 from Germany) with a median age of 22.0 years (range: 13.2–69.7). The molecular diagnoses were *H19/IGF2* LOM in 80.3%; upd(7)mat in 16.9% and *IGF2* mutation in 2.8%. The clinical characteristics are presented in Table 1. There were differences in age at assessment between the different countries (*p* < .001) but other characteristics were comparable.

**Table 1:** Clinical characteristics of participants

	Whole cohort	United Kingdom	France	Germany	*p* Value
Number	71	33	17	21	
*Sex*					
Male	31 (43.7)	15 (45.5)	4 (23.5)	12 (57.1)	.1
Female	40 (56.3)	18 (54.5)	13 (76.5)	9 (42.9)	
Age	22.03 (13.17–69.71)	29.58 (13.36–69.71)	16.59 (13.17–28.50)	21.29 (15.07–29.38)	<.001
*Molecular genetic diagnosis*					
*ICR1/H19* LOM	57 (80.3)	27 (81.8)	16 (94.1)	14 (66.7)	.1
upd(7)mat	12 (16.9)	6 (18.2)	1 (5.9)	5 (23.8)	
*IGF2* mutation	2 (2.8)	0	0	2 (9.5)	
*Growth hormone treatment*					
Yes	55 (77.5)	23 (69.7)	13 (76.5)	19 (90.5)	.2
No	16 (22.5)	10 (30.3)	4 (23.5)	2 (9.5)	
Growth hormone dosage	47.74 (34.85–55.98) (*n* = 46)	48.84 (35.72–54.96) (*n* = 19)	35.71 (30.72–49.29) (*n* = 9)	52.48 (34.70–61.66) (*n* = 18)	.08
*Treatment to delay puberty*					
Yes	20 (28.2)	5 (15.2)	6 (35.3)	9 (42.9)	.01
No	45 (63.4)	27 (81.8)	11 (64.7)	7 (33.3)	
Unknown	6 (8.5)	1 (3.0)	0 (0)	5 (23.8)	

*Note*: Sex, molecular genetic diagnosis and growth hormone treatment presented as number (percentage). *n*, number shown where data for the whole group not available. Age in years presented as median (full range). Growth hormone dosage (mcg/kg/day) presented as median (interquartile range).

Abbreviations: *H19/IGF2* LOM, loss of methylation at the *H19/IGF2* intergenic differentially methylated region; *IGF2*, insulin‐like growth factor 2 gene; upd(7)mat, maternal uniparental disomy for chromosome 7.

### 3.2 Effect of GH on height in the overall cohort

The characteristics of the GH‐untreated and GH‐treated groups are shown in Table 2. GH treatment was received in 77.5% for a median duration of 7.1 years (interquartile range [IQR]: 4.0–11.0). The median value of the mean GH dosage administered was 47.7 mcg/kg/day (IQR: 34.9–56.0) or 1.57 mg/m^2^/day (IQR: 1.13–1.89). There was a suggestion of differences in GH dosages between countries (*p* = .08). The median age of starting GH treatment was 5.80 years (IQR: 4.5–9.0) and the median time since GH discontinuation was 10.0 years (IQR: 2.7–16.0). The GH‐untreated group was older than the GH‐treated group; median ages 28.3 and 21.2 years, respectively (*p* = .03). Total height gain was greater in the GH‐treated group (median gain 1.53 SDS vs. 0.53 SDS, *p* = .007). Early height SDS was lower in the GH‐treated group than the GH‐untreated group (−3.46 SDS vs. −2.91 SDS, *p* = .04). Both groups reached comparable final heights (−2.22 SDS vs. −2.74 SDS, *p* = .7).

**Table 2:** Clinical characteristics of GH‐untreated and GH‐treated groups

	GH untreated	GH treated	*p* Value
Number	16	55	
*Demographics*			
Male	5 (31.3)	26 (47.3)	.4
Female	11 (68.8)	29 (52.7)	
Age	28.33 (19.73–36.97)	21.24 (16.59–27.36)	.03
			
*H19/IGF2* LOM	16 (100)	41 (74.5)	.1
upd(7)mat		12 (21.8)	
*IGF2* mutation		2 (3.6)	
*Growth parameters*			
Early height SDS	−2.91 (−3.62 to −2.40) (*n* = 12)	−3.46 (−5.15 to −2.76) (*n* = 53)	.04
Age at early height measurement	2.69 (2.00–3.76) (*n* = 12)	4.13 (2.28–5.46) (*n* = 53)	.07
Total height gain	0.53 (−0.13 to 1.37) (*n* = 12)	1.53 (0.80–2.52) (*n* = 53)	.007
Final height SDS	−2.74 (−3.36 to −1.13)	−2.22 (−3.66 to −1.16)	.7
Height SDS ≤ −2	9 (56.25)	30 (54.55)	1.0
Height SDS > −2	7 (43.25)	25 (45.45)	
Distance to target height SDS	2.51 (1.76–3.81)	2.30 (1.55–3.01) (*n* = 54)	.5
			
Early BMI SDS	−2.78 (−3.29 to −1.33) (*n* = 12)	−2.65 (−3.81 to −1.91) (*n* = 52)	.3
Age at early BMI measurement	3.37 (2.02–4.03) (*n* = 12)	4.30 (2.20–5.54) (*n* = 52)	.3
Change in BMI SDS	3.58 (1.85–5.18) (*n* = 12)	2.01 (0.76–2.85) (*n* = 52)	.006
BMI SDS at most recent evaluation	1.66 (−0.73 to 2.03)	−1.10 (−1.80 to 0.10)	.002
BMI SDS ≤ −2	2 (12.5)	12 (21.82)	.5
BMI SDS ≥ + 2	4 (25)	1 (1.82)	.008

*Note*: Sex and molecular genetic diagnosis presented as number (percentage). *n*, number shown where data for the whole group not available. Age in years presented as median (interquartile range). Data on growth parameters presented as median (interquartile range). Height SDS ≤ −2, Height SDS > −2, BMI SDS ≤ −2 and BMI SDS ≥ + 2 presented as number (percentage).

Abbreviations: BMI; body mass index; GH, growth hormone; *H19/IGF2* LOM, loss of methylation at the H19/IGF2 intergenic differentially methylated region; IGF2, insulin‐like growth factor 2 gene; SDS; standard deviation score; upd(7)mat, maternal uniparental disomy for chromosome 7.

In the GH‐treated group, those who started treatment before 4 years of age (*n* = 13), were shorter at the onset (median −5.26 SDS vs. −2.98 SDS, *p* = .001), gained more in height SDS during treatment (3.13 SDS vs. 1.42 SDS, *p* = .03) and gained more in height SDS between early and final height (2.57 SDS vs. 1.25 SDS, *p* = .02), compared with those who started treatment later (*n* = 42).

Early height SDS was positively associated with increased final height SDS (*β* = 0.66 [95% confidence interval {CI} 0.51–0.81], *p* < .001) (i.e., those who were taller in childhood were taller at final height) and a similar association was observed with target height SDS (*β* = 0.39 [95% CI 0.09–0.69], *p* = .01) (i.e., those with a greater target height reached a greater final height). Female sex was associated with lower final height. There was a suggestion of an association between molecular genetic diagnosis and height outcomes with cases of upd(7)mat and *IGF2* mutation showing greater final height SDS (*β* = 0.43 SDS, CI −0.04 to 0.90, *p* = .07) (Table 3).

**Table 3:** Multiple linear regression for associations with final height SDS

	Final height SDS		
	*β*	CI	*p* Value
Growth hormone	0.38	−1.04 to 0.27	.2
Early height SDS	0.66	0.51–0.81	<.001
Target height SDS	0.39	0.09–0.69	.01
Female sex	−0.80	−1.27 to −0.34	.001
Epigenotype	0.43	−0.04 to 0.90	.07

Abbreviations: CI; 95% confidence interval; SDS; standard deviation score.

A subanalysis of only individuals with *H19/IGF2* LOM was performed and showed similar results for height outcomes: in the GH‐untreated and GH‐treated groups, final height SDS was similar (median −2.74 and −2.24, respectively, *p* = .9) with greater total height gain in the GH‐treated group (1.47 SDS compared to 0.53, *p* = .02).

### 3.3 Effect of treatment with gonadotrophin analogs on height

GnRHa treatment was given in 19 individuals with a median age at the start of 10.8 years (IQR: 9.6–11.3) (*n* = 19) and a median duration of treatment of 2.3 years (IQR: 1.8–3.1) (*n* = 18). Between the cohorts from countries, there was a difference in the proportion of participants who had received treatment to delay puberty (*p* = .01); however, other features were comparable. There was no significant difference in the proportion of males and females treated to delay puberty (39.3% vs. 24.3%, *p* = .2). In the 20 cases where treatment had been administered to delay puberty, GnRHa treatment had been received in 95% and cyproterone acetate in 10%; one individual had received both treatments. Treatment to delay puberty was not associated with a difference in total height gain or final height SDS in this cohort but numbers are small (data not shown). In all cases of GnRHa treatment, GH was also given.

### 3.4 Effect of GH on BMI

In the overall cohort, GH‐treated and GH‐untreated groups had similar early BMI SDS (median −2.65 SDS vs. −2.78 SDS, *p* = .3). The change in BMI SDS was greater in the GH‐untreated group compared with the GH‐treated group (median 3.58 SDS vs. 2.01 SDS, *p* = .006) and there was a higher BMI SDS at the most recent assessment in the untreated group (median 1.66 SDS vs. −1.10 SDS, *p* = .002). In the GH‐untreated group, 25% (4/16) had a BMI SDS ≥ 2 at their most recent evaluation compared with 1.8% (1/55) in the GH‐treated group (*p* = .008). In the GH‐untreated group 12.5% (2/16) had a most recent BMI SDS ≤ −2 compared with 21.8% (12/55) in the GH‐treated group (*p* = .5) (Table 2).

GH treatment was associated with lower BMI SDS (*β* = −1.89 SDS, CI −3.15 to −0.64, *p* < .004) at the most recent evaluation. Those who started with a higher BMI SDS had a higher BMI SDS at follow‐up (*β* = 0.57 SDS, CI 0.29–0.85, *p* < .001). Sex, molecular genetic diagnosis and GnRHa/cyproterone treatment were not associated with either BMI SDS at the most recent assessment or change in BMI SDS (Table 4).

**Table 4:** Multiple linear regression for associations with BMI SDS at most recent evaluation

	BMI SDS		
	*β*	CI	*p* Value
Growth hormone	−1.89	−0.64 to −3.15	.004
Early BMI SDS	0.57	0.29–0.85	<.001
Female sex	0.16	−0.73 to 1.04	.7
Epigenotype	0.22	−0.74 to 1.17	.6
Treatment to delay puberty	0.08	−0.87 to 1.02	.8

Abbreviations: BMI; body mass index; CI; 95% confidence interval; SDS; standard deviation score

A subanalysis of only individuals with *H19/IGF2* LOM was performed and showed that change in BMI SDS was greater in the GH‐untreated group compared with the GH‐treated group (median 3.58 SDS vs. 1.95 SDS, *p* = .008) and there was a higher BMI SDS at the most recent assessment in the untreated group (median 1.66 SDS vs. −1.09 SDS, *p* = .003).

One individual in the French cohort had been treated with an aromatase inhibitor, however, details of treatment were not obtained.

## 4 DISCUSSION

Our study is the first to compare growth outcomes in GH‐treated versus GH‐untreated molecularly confirmed SRS. Inclusion of only molecularly confirmed SRS cases is important as the clinical features of SRS overlap with other conditions and historical cohorts included those born SGA along with SRS.^11^ Others have included SRS exclusively but included clinical SRS cases with different diagnostic criteria.^8^, ^9^, ^10^ Previous findings may thus not truly reflect the growth outcomes of molecularly confirmed cases of SRS and as we move to an era of personalized medicine, epigenotype‐phenotype correlations are increasingly relevant. We have, therefore, studied a group of older individuals with molecularly confirmed SRS and evaluated differences in height and BMI between those previously treated with GH and those untreated. Such a control group will be increasingly difficult to identify as GH becomes more widely used. This study aimed to evaluate previous treatments to provide evidence to support health professionals' and families' decisions. Our novel findings show that in individuals with molecularly confirmed SRS, prior GH treatment is associated with greater height gain and lower BMI later in life. Greater adult height with GH treatment has been reported in SRS, including molecularly and clinically diagnosed SRS and including home height measurements.^10^ In our study, GH treatment was associated with greater total height gain, providing further evidence in support of GH treatment to enhance height in SRS.

The GH‐treated group was shorter in early life and may have been more severely affected by SRS. Shorter individuals may also be more likely to receive treatment. The final height attained in the GH‐treatment group in our study (median SDS −2.22) was comparable to previous studies in GH‐treated individuals.^9^, ^10^ However, those cohorts included patients with molecularly and clinically diagnosed SRS, whereas our study included exclusively molecularly confirmed SRS.

There was a negative association between age of onset of GH treatment and height gain, similar to a previous study,^10^ suggesting that early treatment initiation should be considered in SRS. Some of the previously reported cases were included in the study reported here.

In our study, females with SRS attained a lower final height SDS than males, in agreement with previous results.^10^ The reasons for this are unclear but it is possible that there is differential sensitivity of the growth plate to sex steroids in men and women with SRS. Sex‐dependent effects of estradiol have been shown on the mouse growth plate,^33^ which would support this theory. There was no difference in the proportion of females and males treated to delay puberty. However, our study did not evaluate height or age at pubertal onset and completion, which would be interesting areas for further research.

We have demonstrated an association between prior GH treatment and lower BMI after treatment. Only 1.8% of the GH‐treated group had a BMI SDS ≥ 2 compared with 25% in the untreated group. GH treatment in SGA has been shown to promote the development of lean mass and reduce fat mass during treatment,^34^ and reduce fat mass but increase central fat distribution.^35^ However, one group has shown increased fat mass SDS during and 2 years after GH treatment in SRS, which was similar to in individuals with non‐SRS SGA^16^ and another study showed a similar fat mass and fat distribution in adults who were born SGA both treated and untreated with GH.^36^ As far we are aware, only one study has evaluated body composition exclusively in SRS but that study was limited to seven cases, the patients were younger than those presented here (mean age of 26.9 ± 9.1 years and range 18–46 years), GH had been received in 2/7 cases and treatment effects were not analyzed.^15^ To our knowledge, no studies have evaluated long‐term BMI after GH treatment in SRS. We speculate that lower BMI results from differences in body composition including reduced fat mass in GH‐treated individuals. GH‐treated individuals may have been more physically active in childhood as a result of their treatment and may have developed beneficial exercise habits that continued into later life. The finding of lower BMI in association with GH treatment is novel and we speculate that treatment may influence a more positive long‐term metabolic outcome.

There were a number of limitations to this study. First, the study was a retrospective, observational study and, although height and weight measurements were performed by a clinician, some data (e.g., birth parameters) were collected from individuals, parents and medical notes and some data was unavailable. Although the proportion of males and females in the treatment groups was similar, information on the ethnicity and socioeconomic status of individuals with SRS was unavailable. Other differences between the treatment groups cannot be excluded. It is possible that recall bias may have occurred among participants, and medical notes were not available in all cases to verify the information. Second, participants were treated at different historical time points and medical practice has changed over this period. Neither the indications for GH treatment nor the protocols used could be established. The GH‐untreated group was older and within the GH‐treated group, there was a wide range of ages, therefore, the treatments reported here may not be representative of current practice. The median GH dosage was also above the current treatment recommendation. The older age of the GH‐untreated group may have influenced the BMI of this group. Individuals with SRS who are untreated are increasingly becoming the exception so the evaluation of this group remains valuable. Third, the clinical practice would have varied between the different countries. Finally, other potential unmeasured, confounding factors could not be examined. For example, there may have been differences between medical teams who administered GH treatment and those which did not. This may have led to better advice about avoiding weight gain in individuals who were born SGA. There were no data available on safety.

Our results suggested that molecular genetic diagnosis (*H19/IGF2* LOM vs. upd(7)mat vs. *IGF2* mutation) impacted final height, but our study was underpowered to assess this. Although there were no differences in rates of GH treatment or its duration, differences were found in additional GnRHa treatment between the countries. Details on the clinical reasoning were unavailable therefore no consistent approach to assessing this treatment could be applied. GnRHa treatment itself was not shown to contribute to final height SDS or total height gain in this study and so we have no evidence that the difference in GnRHa treatment between the country cohorts affected the results. However, previous studies in children with short stature who were born SGA have shown improved adult height with combination treatment with 2 years of GnRHa therapy in addition to GH^37^ and greater height gain.^38^ Greater growth from the onset of puberty to adult height has been shown in girls with clinically diagnosed and molecularly confirmed SRS treated with GnRHa.^9^, ^19^

In conclusion, our data show that in individuals with molecularly confirmed SRS, prior GH treatment is associated with greater height gain and reduced BMI later in life (despite cessation of GH‐treatment many years previously). Lower long‐term BMI with GH treatment may, in turn, indicate an improved prognosis for metabolic health.

## CONFLICTS OF INTEREST

J. H. D.  has received travel bursaries from Pfizer, Ipsen, SANDOZ and Novo Nordisk. G. B. has received honoraria for lectures from Ferring, Ipsen, Lilly, Merck Serono, Novo Nordisk, Pfizer and Sandoz and for membership in advisory boards from Ferring, Ipsen, Novo Nordisk and Pfizer. J. C. assisted with recruitment to the study, providing a family perspective on study design, and reviewed the manuscript in preparation.

## Data Availability

Research data are not shared. Available on request from authors.
